# Exploring Semi-Supervised Methods for Labeling Support in Multimodal Datasets

**DOI:** 10.3390/s18082639

**Published:** 2018-08-11

**Authors:** Alexander Diete, Timo Sztyler, Heiner Stuckenschmidt

**Affiliations:** Data and Web Science Group, University of Mannheim, 68131 Mannheim, Germany; timo@informatik.uni-mannheim.de (T.S.); heiner@informatik.uni-mannheim.de (H.S.)

**Keywords:** machine learning, activity recognition, multimodal labeling

## Abstract

Working with multimodal datasets is a challenging task as it requires annotations which often are time consuming and difficult to acquire. This includes in particular video recordings which often need to be watched as a whole before they can be labeled. Additionally, other modalities like acceleration data are often recorded alongside a video. For that purpose, we created an annotation tool that enables to annotate datasets of video and inertial sensor data. In contrast to most existing approaches, we focus on semi-supervised labeling support to infer labels for the whole dataset. This means, after labeling a small set of instances our system is able to provide labeling recommendations. We aim to rely on the acceleration data of a wrist-worn sensor to support the labeling of a video recording. For that purpose, we apply template matching to identify time intervals of certain activities. We test our approach on three datasets, one containing warehouse picking activities, one consisting of activities of daily living and one about meal preparations. Our results show that the presented method is able to give hints to annotators about possible label candidates.

## 1. Introduction

Many applications in the field of activity recognition rely on labeled data across multiple modalities to build well performing models. A typical example is the combination of inertial data and egocentric video [1]. Proper annotation of data can be time consuming due to the amount of data and, in some cases, the need for knowledge of domain experts. The time consuming factor is mostly rooted in the fact that a video has to be fully watched to be able to find all activities when no prior knowledge about the sequence of activities exists. Thus, automated annotation of sensor and video data is of great interest especially considering the recent developments in wearable devices [2]. With more devices recording data simultaneously, annotation tools can utilize a broader spectrum of data to make their predictions. However, the manual annotation of some amount of data typically goes along with these approaches, as the models need annotated data to be trained properly. Hence, the mentioned problems with annotations still exist for big portions of the annotation process until the model is finally good enough to assist the user. Other existing annotation tools [3,4] often only provide visual support to annotate such data but rarely offer recommendations for labels which speed up the annotation process only marginally. Here an annotator may learn to see reoccurring patterns for activities, enabling him or her to jump though the recording. Still, this requires the annotators to learn these patterns which may take some time unless the knowledge is taught to them in advance.

Therefore, we developed an annotation tool for multi-sensor activity recognition that after a short phase of training is able to assist labeling similar datasets automatically by providing the user annotation suggestions. Our focus in this publication is not the usability of the tool but rather an analysis of methods for label recommendations. Using very few annotations of activities that should be recognized, we can provide annotators with suggestions for possible other new annotations within their dataset. We focus on finding all activities at the cost of precision as we believe that it is easier to correct or discard a label that was assigned a few seconds off the correct position or wrongly instead of finding a missing annotation. On top of that, annotation of data in itself can be very user dependent as agreement on the start and end of activities varies among different annotators which further emphasizes the need for as many labels as possible. Initially, we developed a web-based application which provides support concerning the alignment, analysis, and labeling of inertial and video data. The tool maps the acceleration data onto the video by visualization which enables the user to align and annotate the data. After receiving a small amount of annotations, our tool can provide labeling recommendations. With the visualization and recommendations, labeling-tasks may also be assigned to non-domain experts. For this extension the tool got rewritten to a native application using OpenCV [5] and python TK. The main reason for the rewrite was the better handling of videos on a frame basis which is possible in a web scenario but involves a lot communication with a server that we deemed not fitting for our scenario.

We test our system on different datasets with different activities, ranging from simple (e.g., grabbing an item from a shelf) to complex (e.g., preparing a meal). One scenario we are analyzing concerns order picking in the area of logistics (henceforth called Picking).

In this context, the picking activity means the selection of items from boxes in shelves that make up an order in a warehouse environment. An augmented system can improve this process significantly and therefore we are working on a project that aims to deliver a full augmented picking system that can be used in warehouses without relying on specific barcodes or RFID techniques [6].

For that purpose, we use a custom wristband and smartglasses which collect inertial data and egocentric video respectively. We consider the acceleration data of the band for our simple motion scenario.

A more complex scenario is covered by recognizing Activities of daily living from a dataset we created containing egocentric video and inertial data collected by smartwatches on both wrists. The focus of the dataset is the recognition of hard to distinguish activities which are defined as consisting of similar motions (in our context these were activities that revolve around consuming things like water, food or medicine). Exploiting these similarities, we try to recognize all activities at once. We focused on the subset of activities that were performed while lying down as this offers a contrast to the other activities in the other datasets which were all performed standing up.

Complex activities involve multiple movements and were also analyzed by us. Here we show that our method is able to cluster similar activities based on their motion. We use the CMU-MMAC [1] dataset (Carnegie Mellon University Multimodal Activity) for our analysis as the cooking activities present are full of multiple movements per activity. In the following parts we call this dataset Kitchen.

In this work, we present our method of using acceleration data to recognize activities and create labels to be used for annotating video data. For that purpose, we focus on template matching in the form of dynamic time warping as preceding works already presented promising results [7]. In contrast, a sliding window approach seems to be less promising concerning the recognition across subjects [8]. This work is an extension of our work presented in [9] where we added more experiments, more data and updated the annotation tool to a new version. We are focusing on the question, how well a template matching based approach can be used to recommend labels and an analysis on which methods are working for different datasets. Our contribution is the application of dynamic time warping for recognizing activities among a broad spectrum of data with a focus on aiding manual annotation of data. We also supply an in-depth analysis of factors that influence the results.

This paper is structured as follows: In Section 2, we present the related work. Subsequently in Section 3, we are going to describe the dataset we collected and used for our system. In Section 4, we outline our methods that are used for automatic labeling and the corresponding tool for annotation. Section 5 summarizes our results. Finally, Section 6 describes our conclusion and future work.

## 2. Related Work

Annotation of activities and the quality of the labels is very much dependent on the tools used to annotate data. This was investigated by Szewcyzk et al. [10] who were able to show that with increasing assistance (in the form of visualization and predefined activities) annotators perform a labeling task with higher accuracy and in less time. Therefore, different methods of annotating data (especially videos) have been researched [11,12,13]. Clustering of video information and subsequent visual representation in form of a multi-color navigation was shown to improve the annotation task [12] while methods of browsing videos non-sequentially and in parallel let users grasp the content of a video faster [13]. In many respects though, especially the automatic annotation of video data is challenging [14]. Therefore, many different approaches in automatic annotation have been proposed in previous publications. These are either purely vision based or based on sensor data.

A pure vision based approach was presented by D’Orazio et al. [15] who were able to improve video annotation for soccer games by first applying a pre-trained model to recognize soccer players and afterwards having the annotators correct the misclassified positions. This approach is hard to apply to our scenario though, as the scenarios we consider are located in a more open world setting. For this approach to be feasible, we would need classifiers for a lot of different objects and backgrounds which still may not cover all activities as egocentric video often has the issue of activities only being partially in frame. In addition, our activities are not defined by object occurrence in a frame but rather by the interaction of the user with objects or their environment in general. For that purpose, we focus on the feasibility to transfer automatic recognized labels of acceleration sensor data to corresponding video recordings. Therefore, we focus on template extraction and matching of certain motions.

Such a template based approach was suggested similarly by Margarito et al. [7] who already showed that templates that were extracted from a wrist worn accelerometer sensor are able to recognize certain sports activities across different people. Further, they pointed out that combining different template-matching metrics in context of statistical classifiers could be also promising. Similarly, Martindale et al. [16] used Hidden Markov Models to find annotations and showed good performances for cyclic activities. While the results of these works are promising, the activities that were considered were all cyclic in nature like walking, cycling, and squatting. In contrast, we try to find few, mostly very short activities in recordings with a similar length where we cannot rely on the cyclic property of the activities.

Beside template matching, Spriggs et al. [8] investigated a multimodal based classification approach considering also inertial sensors but adding first-person video data. They focused on daily kitchen activities and performed a frame based classification by relying on features that were extracted from the inertial sensor and video data. However, they clearly state that their approach does not generalize well across people.

Relying only on inertial and force sensors, Morganti et al. [17] stated that already different wrist shapes and muscles configurations across people can affect the recognition procedure. Further, they point out that especially the force sensors they used in their custom wristband enable recognition of specific gestures that could not reliable be recognized by inertial sensors. While their approach is promising, the experiments they presented were in a preliminary state. Moreover, the amount of sensors used in the approach are as of yet uncommon in off the shelf hardware and thus do not integrate with our scenarios.

Focusing on sensor data annotation tools, several researchers already presented powerful and promising annotation tools. However, only few of them provide support concerning labeling recommendation or automated labeling. Palotai et al. [18] presented a labeling framework that relies on common machine learning approaches but was only designed for domain experts. In addition, it is unclear how their approach performs concerning different level of activity types or how different sensors are supported concerning their introduced learning approach (e.g. feature extraction). Indeed, Barz et al. [19] highlight that most data acquisition and annotation tools are mostly limited to a particular sensor. This can be attributed to the fact that it seems to be necessary to consider different techniques or feature sets for different kinds of sensors. Especially the combination of multiple learned models in context of automated labeling seems to be challenging.

It can be seen that for many scenarios, methods for annotating data are differing greatly. Some of them use image features, while others rely on inertial sensors. We therefore analyze one base method and adapt it for different datasets to see, if we can achieve consistent performance among them. Since the datasets cover different scenarios, the adaption of the method is necessary as we even have slightly different tasks per dataset (e.g., finding a single label, finding multiple labels, etc.). For this purpose we use dynamic time warping as it has shown to work on different types of sensor data [7,20,21].

To show an overview, we compare our presented approach to similar solutions and point out the differences in Table 1.

## 3. Dataset

To fully analyze our approach, we consider three different datasets which contain different types of activities, thus allowing a broader analysis of our tool. The first dataset contains activities in the field of logistics (picking scenario). Here we analyzed picking activities [22] that are used to collect items for an order in a warehouse. Each sequence contains one grabbing activity which is always performed while standing in front of a shelf. In the second dataset activities of daily living [23] were recorded, with the activities involving very similar motions. Each sequence contains multiple activities and all of them were performed while lying down. This is also the dataset that was added in this extension. The last dataset we use is the publicly available CMU-MMAC dataset [1] (kitchen dataset). This dataset is the most complex one and involves a variety of activities with different motions.

### 3.1. Picking Scenario

The data recording followed a predefined protocol that contains a sequence of activities, i.e., *walking to shelf*, *locating the correct box*, and *grabbing from the box*. In this context, several scenarios were recorded including picking from different boxes on different rows and from different shelves.

**Figure 1:**
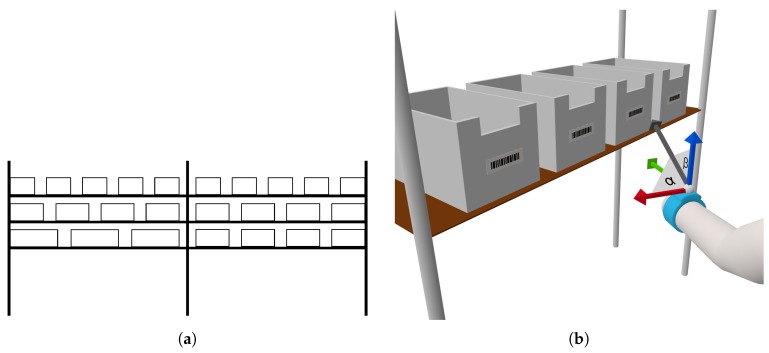
(**a**) Schematic of the shelves in our test environment; (**b**) Angle features from the wristband used for matching.

The test environment consists of two shelves located next to each other where each shelf has three rows of boxes with three to five boxes per row. Thus, the boxes were placed on different heights and were spread horizontally among two shelves (see Figure 1a). The test environment is setup based on a real warehouse that we could inspect in regard of a company project. A problem with recognizing grabbing motions is the variation of that activity, thus, a grabbing motion can produce highly different sensor outputs depending on the location of the object to be grabbed. In contrast, activities like walking or running do not have this degree of variation. Therefore, our dataset contains multiple different cases of grabbing within the shelf to cover the space of different motions.

**Figure 2:**
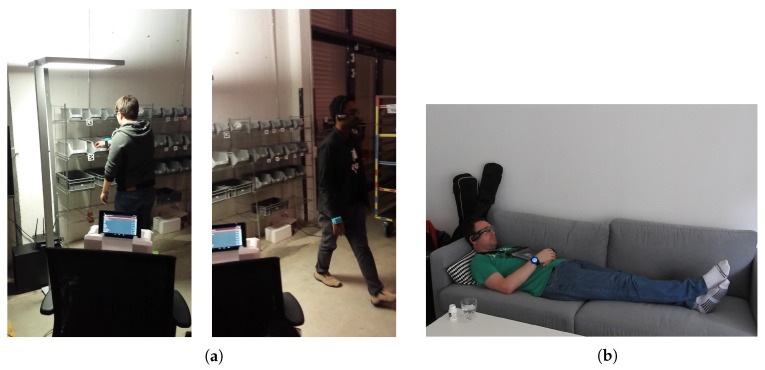
Photos from the recording processes of our datasets. (**a**) Picking scenario. Participant walking towards and away from shelves; (**b**) ADL scenario. A glass of water and pillbox on the table.

The required data for our dataset was collected using smartglasses (Vuzix M100, Android 4.0) and a custom wristband. Both devices recorded acceleration, gyration, and magnetic field data while the smartglasses also recorded video information of its front camera. The sensors of the wristband are all read at the same point in time while the sensors of the smartglasses cannot be recorded synchronously (Under Android, sensors are not queried but the system pushes new values. Hence, there is no guaranty concerning the specified frequency). Further, as the wristband and the smartglasses are not connected, the recorded timestamp of the data has to be synchronized afterwards. For that purpose, the subjects were instructed to stand idle for a period of time before and after the performance of the activities. Data on the wristband was collected at 40 Hz for all the sensors while the smartglasses recorded the sensors at 50 Hz and 25 fps respectively. Hence, we recorded with the highest possible frequency. For better interpretation, each recording session was also filmed from a third-person-perspective (see Figure 2). This enables also to collect depth information of the recorded images in form of point clouds.

For the recording, we relied on a self-developed application. Thus, we enhanced an Android application of a previous work [24] where especially the support of smartglasses was added. The recorded data of the smartglasses is stored locally on the device. However, the custom wristband does not have enough storage to store the data locally; hence, we had to send the recorded data directly over Wi-Fi to a server.

### 3.2. Activities of Daily Living Scenario

We also recorded an additional dataset that contains activities of daily living. In total, we collected data for two participants who performed multiple activities per recording lasting between one and three minutes with a maximum of four activities per recording. This dataset focuses on activities that can be hard to distinguish due to similar motions. Specifically, we looked at food, water and medicine consumption which all include motions of reaching towards an item and then consuming it orally. The data was collected similarly to the previous example but exchanged the custom wristband with a smartwatch (and a corresponding smartphone that was located in the test subject’s pants pocket). Also, we added another watch on the non-dominant hand, enabling us to capture motions from both wrists of the test subject. In addition, we used the aforementioned tablet as a chest mounted camera recording another perspective of egocentric video without collecting depth information. In total, we collected data from six devices: IMU from both phones, both watches, the tablet and the smartglasses and video from the smartglasses and the tablet. We also recorded the whole test scenario from a third-person-perspective to make annotation easier. For this work we looked at three different activities: eating prepared food from a plate, taking medicine and drinking water.

### 3.3. Kitchen Scenario

As an additional reference, we also considered the dataset *CMU-MMAC* which was published and described in great detail by F. Torre et al. [1]. In contrast to our own datasets this one is far more complex in multiple ways. Hence, the dataset contains more different arm motions e.g. getting a cup from a shelf includes opening the shelf and grabbing the cup. The motions themselves are also not as homogeneous as they are in our dataset since the setting of a kitchen leads to many different arm movements for retrieving objects. Like in our activities of daily living scenario, they also recorded the movement of both arms instead of just one. However, here we could not assume the dominant hand of the participant confidently.

In our experiments, we consider both cases, i.e., simple and complex, to clarify the feasibility and performance of our approach. In this context, we consider a subset of the *CMU-MMAC* dataset. In particular, we only looked at one recipe, i.e., the brownie recipe, for a subset of all the participants because it was the only one which was completely labeled.

## 4. Method

The method section is divided into three parts, each dealing with one of the aforementioned datasets. Since the datasets are differing in the type of activities, we are presenting methods for each dataset. Methods for the picking and the CMU-MMAC dataset were already published in [9]. The methods for the ADL dataset are new and an extension of the methods for the picking dataset. We first present the findings of the picking dataset, afterwards the extension for the ADL dataset. At the end we present methods for the CMU-MMAC dataset which differ from the previous two methods as they are more concerned with finding similarities among complex activities. All methods are dealing with our goal of offering label suggestions towards user and do not contain aspects of usability within annotation tools.

**Figure 3:**
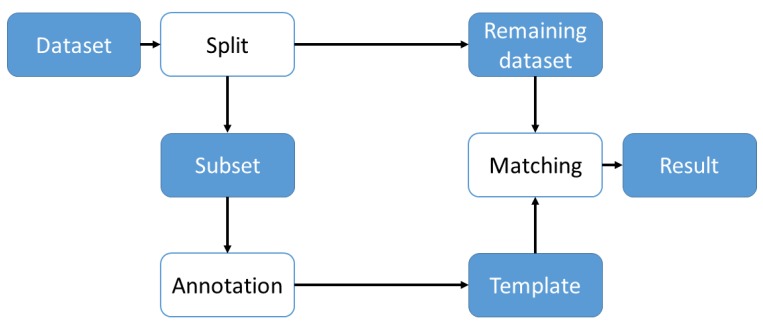
Basic approach for finding matches in a dataset. Blue boxes represent data, white boxes the processing of data.

Figure 3 shows the basic approach used to find matches in a dataset. We label a subset of data and use these labels to create templates that are used for matching in the rest of the dataset. The approach for each dataset will be described in the following subsections. As we described in Section 2, methods for matching may have to be adapted when considering different data. Therefore, in an application the specific method has to be chosen based on the data that has to be annotated. Regarding our own dataset we also used similar approaches to align our data which was recorded with non-synchronized devices. Both datasets were recorded with a third person camera for labeling purposes and contain distinct points in time that enable the alignment of the data. To align the data, we first annotate the distinct motion in the third person video and, for the evaluation later, the labels we want to find. Then, we plot the acceleration data we want to analyze and find the same distinct motion in our data. Once both points in time are found, we can then map the labels from the video to the acceleration data and validate its position in time. For this purpose, we display the labels in time as an overlay of the acceleration plot and therefore can see if the labels are correct. As our datasets do not contain individual recordings that are longer than five minutes and sampling rate of sensors is stable, drift of time and delay in transmission are negligible.

### 4.1. Picking Dataset

**Figure 4:**
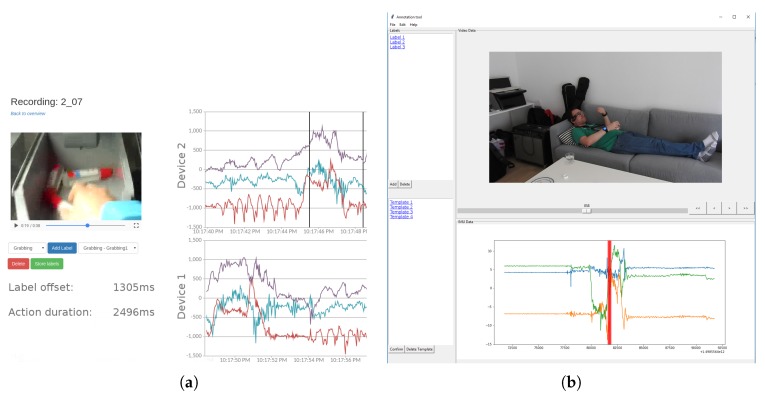
Developed labeling tools. First version running in a browser, second version as a standalone application. (**a**) Old web application; (**b**) New desktop application.

As a first step, we align our picking data in respect of the timestamp where we considered zerolines (a period of no movement) at the beginning of each recording that allow us to pinpoint the starting time of an activity. More precisely, we used the peaks of walking motions in accelerometer plots to align the data as those were easily identifiable as the first activity. This is in line with the process described in the introduction of this section, with the walking being the distinct motion for alignment. We were considering an alignment pipeline for the tool but since alignment methods may vary among datasets alignment information has to be created externally. Subsequently, we labeled the grabbing activity by analyzing the acceleration sensor data that represents the motion and countercheck against the video data that describes the same time period. This allows to label all sensor recordings simultaneously. Once the boundaries of an activity are defined, the application replays the corresponding part of the video that were recorded from the smartglasses (see Figure 4). After the confirmation of the correctness, the corresponding acceleration sensor data is extracted for creating a template of this activity where a template is represented by start and end timestamp, the corresponding acceleration data, and a label.

For now, we focus on the acceleration data because preliminary experiments have shown that the angles relative to the three axes (Section 3, Figure 1) are promising concerning the characterizing of the grabbing motion in context of a wristband. After a certain number of templates of the same activity are available, we apply dynamic time warping [25] to identify possible matches. We assume that the same motions produce similar outputs which only differ in respect of their length due to the varying speed the activity was performed. Thus we chose dynamic time warping as it allows us to match time series of different lengths. Dynamic time warping works by finding a path between two time series that have the smallest distance. The minimal distance is found by first initializing the distance from every point in series A to the first point in series B to infinity and vice versa. Afterwards the algorithm iterates over the combination of all points in both series and calculates their distance by using a cost function (in our case Euclidean distance). The function compares single points and the cost of the path leading to the previous points (recursive):

d(i,j)=cost(i,j)+min(d(i,j−1),d(i−1,j),d(i−1,j−1))

By considering three preceding options that lead to i,j, the algorithm can cope with different lengths of series. The extracted templates slide over an unlabeled dataset to detect the time when an activity occurs. In this context, we try to find the position of the template with the smallest deviation while assuming that at least one activity occurs in the unseen data.

### 4.2. Activities of Daily Living Dataset

**Figure 5:**
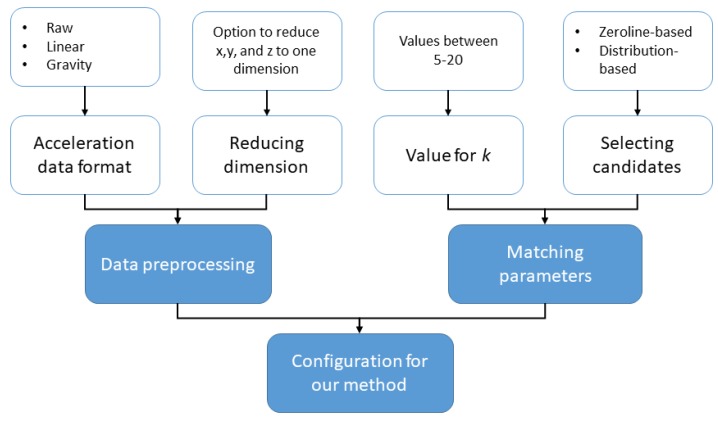
ADL experiment settings. Each parameter is set for a specific configuration of the matching algorithm.

For our activities of daily living dataset, we considered a small subset of activities where the test subject is lying. By looking at the human body pose of lying, we broaden the scenarios that we analyze in our experiments. For the alignment of the data we used zerolines in the acceleration data. We considered the moment the participant starts their first activity as we do not have long walking distances which can be used to align peaks. After alignment, the plot of the data with markers for labels was consistent and showed labels at the correct point in time. In contrast to the picking scenario, the dataset of our ADL scenario has multiple activities per run. Therefore, we cannot pick the best match but rather try to find a set of best matches. Due to the similarity of the activities, we were not trying to match the activities themselves but rather the sub-activity of raising an arm and reaching towards a glass of water, a plate, or a pill bottle. This motion is similar to the picking motion but contains more variation as the environment is more dynamic. Afterwards, a person labeling the dataset can manually distinguish which activity to assign to the template matching results. By applying this trade-off we minimize the amount of pre-labeling for the user as we do not have to have templates for each activity. Methods used in the picking dataset were extended and new ways of transforming the data as well as evaluating it were added. Figure 5 shows the configurations which we tested with our new methods. They can broadly be classified into parameters that influence the input data format for our algorithms and different settings for selecting candidates for our final results.

For the preprocessing of the acceleration data we consider two parameters that can be set. One parameter is what type of acceleration is used (linear, gravity, or raw data), the other parameter is an option to reduce the three axis of acceleration data into one.

- **Acceleration data type**. We distinguish between two ways of transforming the acceleration data we collected. An option is to either use the linear acceleration or gravity of the data by applying a low-pass filter. We also test the raw data collected by our Android application to match the activity.
- **Reducing dimensions**. This option specifies whether the acceleration in the x, y and z dimension should be reduced to a single value. In preliminary tests for the data we could often see that matching templates with only one dimension of data yields better results. One reason for this improved results may be the loss of orientation-information when reducing dimensions which may yield a more generic model. We reduced the dimensions by interpreting the x, y, z acceleration values as a vector and calculating its vector length.

Once the data has been transformed we apply dynamic time warping and then select $top_{k}$ candidates. Therefore, our method also has two configurations for the candidate selection: the *k*-value in our $top_{k}$ selection and the method how we select the candidates.

- **Values for** k. As we are considering a set of activities per recording, we retrieve the ${\mathrm{top}}_{k}$ best matches. For the evaluation we tested values for k ranging from 5 to 20.
- **Selecting candidates**. We apply two different methods for picking the best matches since only choosing the points in time with the lowest distance do not yield the best results. Each method is described in more detail bellow.

After deciding on the parameters, we match the data with the dynamic time warping algorithm. In this case, we use the subsequence matching variant of dynamic time warping as described in [26]. To get candidates for our labels, we first match the template against the data. Then we explore two variants of picking potential matches for our activity. In the first method we match a zeroline template against the data as well. We assume that the distance of a zeroline template is smaller than the distance of the proper activity we try to find in cases that are not connected to the activity. This way we reduce the amount of candidates we have to consider. For all matching candidates we take the $top_{k}$ matches with the smallest distance and return them to the program. In our second method of finding candidates we use another simple assumption. When considering candidates, we look at the current selection we made. A candidate for a match is only added to the list of $top_{k}$ candidates if it is not within a distance δ of any currently selected $top_{k}$ candidate. We set δ to two seconds as our activities are not performed within a shorter period of time. Both methods for selecting candidates can be seen in Algorithms 1 and 2.

Once the candidates are determined, we evaluate them based on the distance to the actual labels.


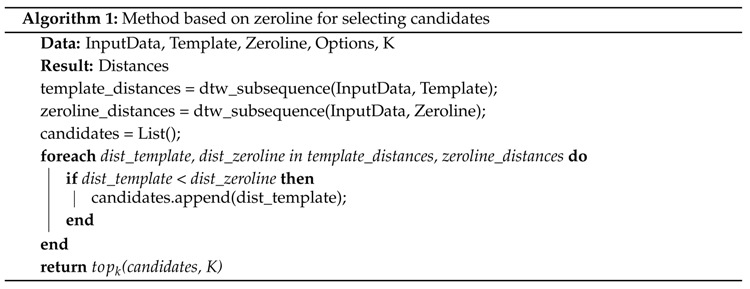



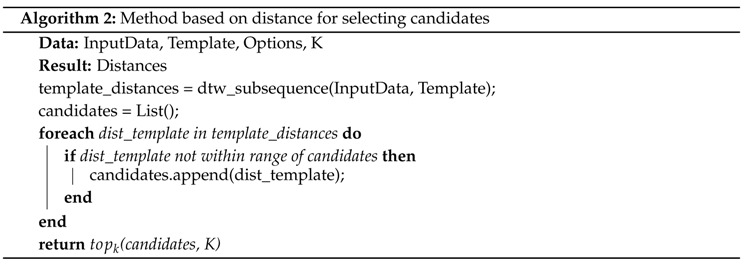


### 4.3. Kitchen Dataset

Focusing on the *CMU-MMAC* dataset, due to its complexity, we have to consider different steps. Hence, in contrast to picking, people switch between the left and right hand which means that it is also necessary to identify which hand was used for the current activity. In our ADL dataset we also had the case of using both hands for activities but we could mitigate that uncertainty by additionally annotating the hand which performed the activity and then matching the activities separately. This dataset already provides aligned data. Thus no further work from our side was needed. Therefore, we unify the data of the same sensor type of both hands so that the current activity is represented by a single vector. Considering the corresponding labels, it stands out that the described activities cover several motions, e.g., grabbing is only a sub-activity. Therefore, still focusing on acceleration data and considering the corresponding ground truth to extract the templates, we segment the data of a template into small windows to compute features that have a stronger expression concerning more complex activities. This includes the used energy (Fourier transform) and median absolute deviation. Due to these high-level labels, several different activities may cover common sub-activities, e.g., taking a pot or turning on the stove includes grabbing. Therefore, we also investigate if the extracted templates have a label independent correlation. We assume that the extracted templates could be grouped to activities that are specific in their motion and not in their semantic. For that purpose, we apply agglomerative clustering to group the templates where the distances between the clusters are the result of the dynamic time warping. Detecting the motion similarity between certain activities, may allow to generalize activity labels but also to construct more robust templates due to the varying executions which in turn support to avoid overfitting.

For the experiments, we perform leave-one-out cross validation. Thus, extracted templates from *n*−1 datasets, and applied them on the remaining one.

## 5. Experimental Section

In this section, we focus on the performance of our labeling support tool to see if it is a feasible approach to be used in greater scale. This involves an evaluation against ground truth data to establish, how well the tool is able to find labels. Since we considered different methods for our datasets, we also evaluated them differently. We evaluate our approaches mainly in regards to how close our estimated label is located to the correct label. A value that describes how far close the estimate was towards the correct label (in our case called delta) is more in line with the task. To give readers an intuition of the results we still added measures for recall for appropriate experiments.

### 5.1. Picking Dataset

In our first experiments, we only focused on the grabbing activity in context of the acceleration sensor data that correspond to the wristband. Thus, we want to investigate the feasibility to apply template matching across different people to identify certain activities where in turn the result should be used to provide recommendations concerning the labeling of the video recordings.

For that purpose, we first applied our introduced method on our picking dataset. We extracted the grabbing motion templates from all except one dataset where each set covers a complete picking process. Then we measured the temporal overlap of the estimated and the actual grabbing motions. For the average overlap per dataset we took the best match (i.e., the match with the least distance) for each template. Afterwards, we selected the most promising subsets of matches and used them to calculate the average overlap for each test dataset. The most promising subset of matches is determined by evaluating all the subsets of the match results with *k* elements and then selecting the one with greatest overlap among itself. Empirical results showed that a value of *k* = 6 yielded the best results in our dataset. Table 2 summarizes the results and points out that we were able to detect nearly all grabbing motions but have an issue concerning the accuracy of the start and stop boundaries. Indeed, inspecting the individual results strikes that the assumption that the searched activity has to have the same length as the considered template leads to an inaccuracy.

**Figure 6:**
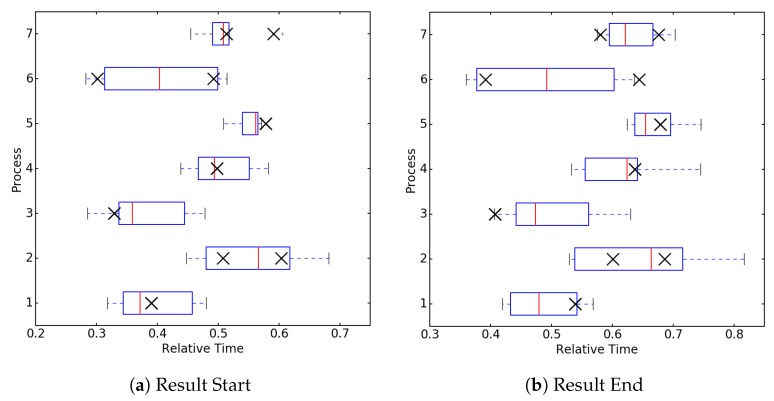
Overall estimate of grabbing start (**a**) and end (**b**) point for picking dataset. Cases 2, 6, and 7 contain two activities and therefore also two crosses in the plot.

Figure 6 describes in detail the recognition and distribution results for all start and stop timings. We want to emphasize that the x-axis does not represent the recognition rate but the relative duration of the whole process. Hence, the box plot represents the time interval were we suppose the start point, respectively the stop point, for the activity that should be recognized. Every box represents the best match for the templates where the x markers show the actual point in time of the grabbing motion. As there can be two grabbing motions in a dataset we plotted both positions. The boxes provide an interesting insight concerning the reliability, i.e., most of the extracted templates were able to identify the correct area of a certain activity across different recordings of the same process.

### 5.2. Activities of Daily Living Dataset

For our activities of daily living scenario, we evaluated different settings of the algorithm. Within the evaluation we first consider the $top_{k}$ best matching results with *k* = 10. After finding the best pre-processing settings, we further tested different values for *k*. We compare different types of pre-processing the data as well as picking best matching candidates. Data can either be used unchanged, or be transformed to get linear acceleration or gravity information. For each of these types we also compare the performance of using all three axes of the data or reducing it to one dimension. Finally, we pick best matches by considering one of two options. One option looks at all the matches that have a smaller matching distance value than the zeroline template at the same point in time. Of those matches the k smallest values are chosen. The other option is picking lowest matching distance values that also cannot lie within two seconds of each other (see Section 4 and Algorithms 1 and 2). All distance values are calculated using the matching results with ten different templates and then summing the distances. The results can be seen in Table 3.

We can see that the best performing combination of settings is the usage of linear acceleration with the dimensions reduced to one and using a delta-based $top_{k}$ approach with a median error of 0.8 s and 0.5 s for both subjects. It can also be seen that the approach of using the zeroline for choosing possible matches is not performing as well as the delta rules-based approach. It even does not return values for some of the use cases, namely the cases that do not reduce the values to one dimension. It seems that a zeroline returns a smaller distance over all the datasets. Further analysis of the approach will be done just on the best performing setting of using linear data with the dimensions reduced to one and using a delta rule-based candidate selection.

**Figure 7:**
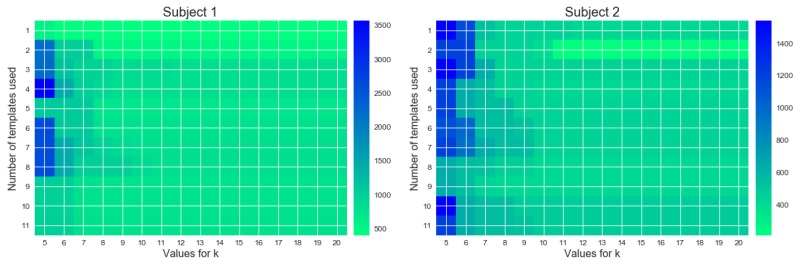
Results of matching activities of daily living with different numbers of templates used for matching and different values for *k*. The color shows the average distance (in ms) of a match to a label.

Figure 7 shows the results of different evaluation parameters with before mentioned best performing methods of data transformation and candidate choosing. It can be seen that the amount of templates used to find matches is not affecting the results significantly. Rather the chosen k is more important to get a reasonable result. It can be seen that just using two templates and then picking the $top_{10}$ matches is sufficient to find activities within a reasonable margin of error.

To further evaluate the performance of our method and provide another form of intuition, we also show recall and ROC curves for the results. The performance is split up for each hand separately in this scenario, to show the differences in the results.

**Figure 8:**
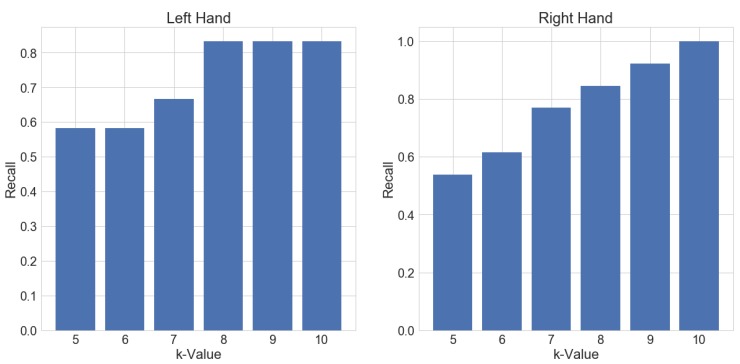
Recall of the results of both hands depending on the value of *k* that was used for candidate selection. An overlap with the ground truth labels is counted as a True Positive.

**Figure 9:**
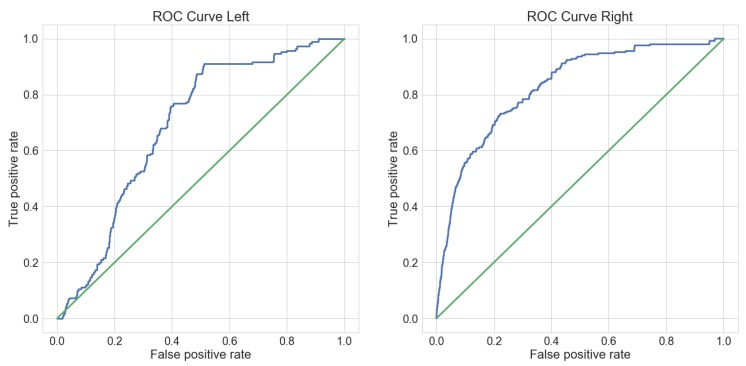
ROC curve for both hands. Without the candidate selection, this plot shows the overall performance of the Dynamic Time Warping algorithm. Again it can be seen that the performance for the left hand data is not as consistent as the performance of the right hand data.

The plot in Figure 8 shows how the recall for each separate hand is changing with different values for *k*. It can be seen that recall for the left hand is behaving differently than the right hand evaluation. The ROC curve in Figure 9 also reflects this fact. This is most likely due to the fact that the primary hand of the subjects is the right hand. Therefore, the motion of the left hand is not as consistent as the motion of the primary hand. Overall it can be seen, that with appropriate values for *k*, the method yields acceptable results for labeling purposes.

### 5.3. Kitchen Dataset

**Figure 10:**
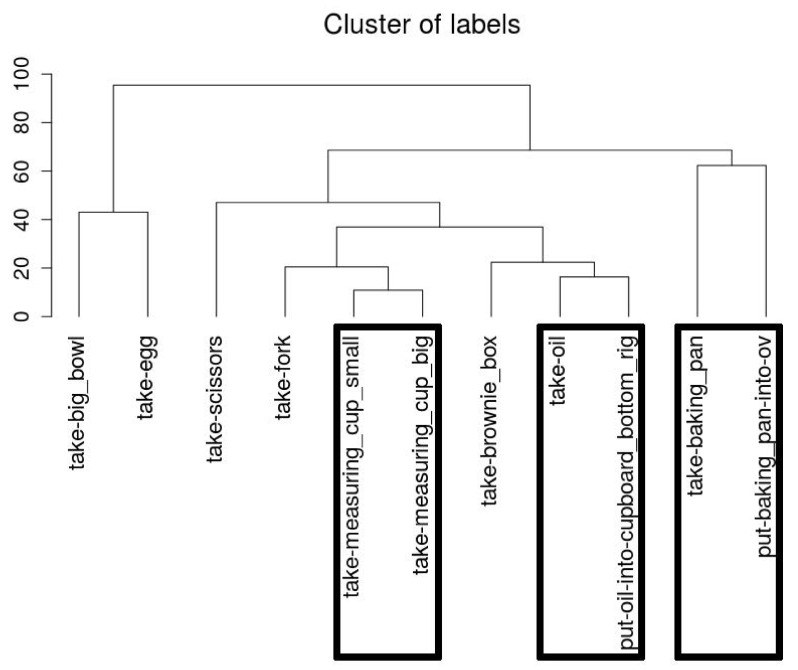
Dendrogram of the clustering of the templates in the kitchen dataset. Marked boxes are activities using the same item.

Considering the *CMU-MMAC* dataset, our first results were insufficient because different activities covered similar arm movement. For instance, the extracted templates of the activity *take oil* also recognized *put oil into cupboard*. Thus, we tried to cluster the activities based on their similarity to get an insight regarding their meaning. Figure 10 illustrates the clustering result of one sample set. It is striking that some activities that use items within a similar location are ending up in the same cluster fairly consistently. For instance, we can observe that motions like taking the big and small measuring cup are very similar. However, in contrast the fork and the scissors for instance are both located in a drawer but end up in the same cluster fairly late. We believe that this is most likely due to fact that the activities are more variable in length than they are in our own datasets. Even though dynamic time warping is able to handle different lengths of time series it is still very likely that the distances of short templates are generally smaller and thus end up faster in clusters than the longer activities like for instance taking the baking pan from the oven.

## 6. Conclusions and Future Work

In this work, we investigated the possibility of a smart data annotation tool that provides labeling recommendations based on already labeled acceleration sensor data. These recommendations can be used to annotate a video and/or acceleration data. We aim to reduce the labeling effort but also want to determine to which extent the recognized labels from the acceleration sensor data could be used to label and process video recordings. The focus was put on recommending labels to annotators that could greatly reduce labeling effort. For that purpose, we performed experiments to investigate the feasibility of applying template matching in context of dynamic time warping to recognize certain activities across different processes and people. In this context, we focused on acceleration data of a wristband and smartwatches to recognize certain activities. It has emerged that it depends on the granularity of the considered activity labels which recognition technique is promising. Hence, activities that actually consist of several sub-activities may have to be considered initially separately. To further investigate this we adapted the experiments to another dataset, containing activities of daily living. Here, we looked at activities that involve eating, water consumption and medicine intake. We could show that these activities, which all involve similar movements, could be recognize fairly consistently by applying template matching and only matching the initial arm movement part of the activity. In this context, we also showed that clustering existing templates from a labeled dataset allows to infer similarities in motion from semantically different activities. This can be considered as a starting point to construct more robust templates while the clustering results also yield more information for a specific motion which in turn reduces the need to perform a certain activity more frequently to get enough characterizing information. Besides, in contrast to other approaches [8,14], we need significantly less data to guess the correct time frame of a specific activity.

In our future work, we want to focus on the problems which came up during our investigations. This includes the recognition quality of the boundaries of activities due to the limitation of a predefined template length but also that we considered so far only acceleration data to extract labeling recommendations. Thus, considering further sensors may also increase the recognition accuracy. For that purpose, we want enhance our own dataset concerning the number of instances but also regarding the considered activities since it turned out that the considered activity level is essential. Another important step in the future work is a user study with a group of annotators that measures annotation time as well as agreement for a set of labeling task with and without the recommendations of our proposed solution. Results from such a study could also point us to other improvements of our method that we have not considered yet.

## Figures and Tables

**Table 1:** Comparison of different annotation tools with their different focuses.

Approach	Idea	Method	Pros and Cons
Our approach	Suggest labels based on a small subset of annotations. In depth analysis of different preprocessing methods and variants of dynamic time warping.	The main focus is the analysis of different variants of dynamic time warping used for label-, and clustering-suggestions. The methods were applied to wrist worn sensors mostly. Evaluation was done in regards to time offset to the correct label and recall of the method.	An in-depth analysis among different datasets with different configurations. On the downside the tool itself is just a prototype, usability of the tool is not tested.
Label Movie [18]	Designing a complete multimedia annotation tool with automatic annotation and crowd-sourcing capabilities.	Used dynamic time warping and SVM time series prediction with a focus on usability of the application. Classification results shown in a Gram matrix to the user. Focus on crowd-sourcing capabilities with the combination of domain experts’ and technical experts’ knowledge.	The tool is fully developed with a lot of functionality, especially the capability for crowd-sourcing. On the downside the evaluation of the tool is lacking in detail and it is not publicly available.
Multimodal Multisensor Activity Annotation Tool [19]	A multimodal annotation tool that is able to handle multiple sensor types like video, depth, and body worn sensors.	The focus is put on capturing many different types of sensors and displaying them in a useful fashion. In contrast to the other methods, this tool is able to capture sensors live and synchronize them. Capabilities for automated annotation are present, but not implemented yet.	Live capturing of different types of sensors is integrated and the tool seems to be designed very concisely. However, as of yet automatic annotations are not integrated though the architecture allows for that.
Smart Video Browsing [12]	Using clustering methods, automatically segment videos into different parts to improve navigation within a video.	For clustering the tool uses color and motion features to distinguish different parts of the video. These can be browsed by the user to distinguish different parts of the video.	The tool does not rely on pretrained methods and can thus easily be used. It does not, however, provide automatic labeling functionality.

**Table 2:** Recognition performance of template matching for picking dataset. The overlap (avg. 69%) is excluding outliers and represents only the best match within a dataset. Cases 2, 6, and 7 contain two grabbing activities.

Dataset	1	2	3	4	5	6	7
**Overlap**	0.43	0.67	0.78	0.52	0.72	0.74	0.99
**Motion [s]**	5.02	2.49	2.55	4.23	2.86	2.43	2.04
		2.22				4.11	2.60
**Δ Start [s]**	1.41	1.89	0.91	0.86	0.71	2.88	0.65
		1.81				2.61	2.91
**Δ Duration [s]**	1.65	0.74	1.40	1.46	0.63	0.68	1.99
		0.68				1.52	1.43

**Table 3:** Results for matching activities of daily living. For each case we report min and max distance to activities and median distance. The bold values show the best results.

Acceleration	Reduce dim.	Approach	Subject 1	Subject 2
Raw	Yes	Zeroline	[0 s - 28.9 s] $\tilde{x}=14.7$ s	[0 s - 39.3 s] $\tilde{x}=7.9$ s
		Delta	[0.2 s - 5.1 s] $\tilde{x}=0.6$ s	[0 s - 1.4 s] $\tilde{x}=0.5$ s
	No	Zeroline	∅	∅
		Delta	[0.1 s - 14.5 s] $\tilde{x}=2.2$ s	[0 s - 39.3 s] $\tilde{x}=7.7$ s
Gravity	Yes	Zeroline	[0 s - 20.1 s] $\tilde{x}=10.2$ s	[0 s - 39.3 s] $\tilde{x}=11.1$ s
		Delta	[0 s - 1.5 s] $\tilde{x}=0.27$ s	[0 s - 12.5 s] $\tilde{x}=0.8$ s
	No	Zeroline	∅	∅
		Delta	[0.2 s - 10.9 s] $\tilde{x}=1.6$ s	[0.2 s - 41.3 s] $\tilde{x}=3$ s
Linear	Yes	Zeroline	[1.2 s - 82 s] $\tilde{x}=24.2$ s	[0 s - 48.4 s] $\tilde{x}=8.2$ s
		**Delta**	**[0.1 s - 1.5 s] $\tilde{x}=0.8$ s**	**[0 s - 1.4 s] $\tilde{x}=0.5$ s**
	No	Zeroline	∅	∅
		Delta	[0.4 s - 9.6 s] $\tilde{x}=2.2$ s	[0 s - 21 s] $\tilde{x}=3$ s
